# Incremental Prognostic Value of Admission Blood Glucose to Albumin Ratio in Patients with Acute Coronary Syndrome: A Retrospective Observational Cohort Study

**DOI:** 10.31083/RCM26567

**Published:** 2025-04-24

**Authors:** Maoling Jiang, Qiang Chen, Qiao Feng, Xiufen Peng, Juan Liu, Hui He, Hong Su, Dongyue Jia, Lin Tong, Jing Tian, Shiqiang Xiong, Lin Cai

**Affiliations:** ^1^Department of Cardiology, Affiliated Hospital, Southwest Medical University, 646000 Luzhou, Sichuan, China; ^2^Department of Cardiology, The Third People’s Hospital of Chengdu, 610014 Chengdu, Sichuan, China; ^3^Graduate School of Peking Union Medical College, Chinese Academy of Medical Sciences and Peking Union Medical College, 100730 Beijing, China

**Keywords:** GRACE score, admission blood glucose, albumin, percutaneous coronary intervention, acute coronary syndrome, prognosis

## Abstract

**Background:**

Blood glucose and serum albumin can be biomarkers at admission since they are easily accessible and demonstrate correlations with cardiovascular diseases. The predictive ability of the admission blood glucose to albumin ratio (AAR) for long-term prognosis in patients with acute coronary syndrome (ACS) and its potential to elevate the predictive value of the Global Registry of Acute Coronary Events (GRACE) risk score in ACS patients post-percutaneous coronary intervention (PCI) remains unknown. Hence, this study aimed to investigate the incremental prognostic value of the AAR in patients with ACS undergoing PCI.

**Methods:**

A rigorous development-validation approach was implemented to optimize the GRACE risk score, utilizing the AAR parameter in 1498 patients suffering from ACS after PCI at the Third People’s Hospital of Chengdu, Sichuan, China.

**Results:**

Over a median of 31.25 (27.53, 35.10) months, the incidence of major adverse cardiac events (MACEs), defined as a composite outcome encompassing all-cause death, cardiac death, nonfatal myocardial infarction, nonfatal stroke, and unplanned repeat revascularization, was higher in individuals with higher AARs. Thus, the AAR was an independent predictor of long-term prognosis in ACS patients undergoing PCI (HR, 1.145; 95% CI: 1.045–1.255; *p* = 0.004). The integration of the AAR score with the GRACE risk score increased the C statistic from 0.717 (95% CI: 0.694–0.740) to 0.733 (95% CI: 0.690–0.776) (*p* < 0.01).

**Conclusions:**

The AAR is an independent predictor of prognosis in ACS patients and significantly increased the predictive value of the GRACE risk score.

## 1. Introduction

Coronary heart disease (CHD) remains the primary cause of mortality worldwide [1, 2]. The Global Burden of Disease study (GBD) showed that the age-standardized rate of deaths for CHD was 108.7 (99.8 to 115.6) per 100,000 individuals, and remains expected to be the leading cause of death through 2050 [3]. Acute coronary syndrome (ACS) makes an important contribution to the mortality of CHD, the crude mortality from ACS was 42.0 (24.7–56.2) per 100,000 people in males and 26.8 (15.0–40.6) per 100,000 people in females, which accounted for 23% (male) and 18% (female) of the total deaths from cardiovascular diseases up to 2020 [4]. Early and timely risk stratification is of utmost importance for the treatment and management of ACS patients. In recent years, the Global Registry of Acute Coronary Events (GRACE) score has been a wide risk stratification tool for ACS patients across international guidelines [5]. However, there was a lack of some important admission biomarkers such as glucose and albumin, which have been shown closely correlated to ACS [6, 7, 8, 9]. Consequently, the GRACE score may not fully capture the intricate condition of ACS patients, to date, the area under the receiver operating characteristic (AUC) curve ranged from 0.70~0.80 [10, 11, 12, 13, 14], but there is still an opportunity to improve it. Therefore, it is critical to research whether the integration of additional novel biomarkers with the GRACE risk score could provide a more precise and reliable risk assessment tool.

Stress hyperglycemia on admission is a significant contributor to inflammatory and oxidative stress responses, which can lead to significant impairment of coronary blood flow, enlargement of infarct size, and acceleration of the progression of ACS [15, 16, 17]. Stress hyperglycemia has been shown to worsen the prognosis of ACS and is correlated with an elevated risk of adverse cardiovascular outcomes [18, 19]. Moreover, another important metabolic indicator of the body is albumin, which is a protein with multiple functions associated with diabetes mellitus (DM), thrombosis, and inflammation [20]. Changes in albumin levels are indicative of systemic inflammation and nutritional status, low albumin levels, often reflecting either malnutrition or heightened inflammatory responses [21, 22]. Previous research found that low serum albumin level on admission is not only a trigger for CHD [23], but also closely correlated with adverse outcomes in ACS patients [24, 25]. The associations between stress hyperglycemia, albumin levels, and cardiovascular prognosis underscore the importance of comprehensive metabolic assessment in ACS patients. In initial research by Zhen* et al*. [26], the AAR was an independent predictor in patients who experienced ST-segment elevation myocardial infarction (STEMI), the AAR was higher, the in-hospital all-cause mortality and the adverse prognosis was higher. However, the primary endpoint in Zhen’s study was in-hospital all-cause mortality, and their median follow-up time was just 1.66 years, the predictive ability of long-term out-of-hospital MACEs in ACS patients remains unknown. Additionally, their study population was STEMI patients [26], the predictive ability of patients with unstable angina (UA) and non-ST-segment elevation myocardial infarction (NSTEMI) remains unknown. To date, it remains uncertain the predictive value of AAR in ACS patients and whether adding AAR to the GRACE score improves the predictive value for long-term prognosis.

In this study, we included all ACS patients, aimed to investigate the predictive value of the AAR for MACEs in ACS patients undergoing percutaneous coronary intervention (PCI) and explored whether there was an underlying incremental prognostic value of the AAR for the GRACE score in the prediction of prognosis.

## 2. Materials and Methods

### 2.1 Study Population

Between July 2018 and December 2020, a total of 1886 patients hospitalized at the Third People’s Hospital of Chengdu (Sichuan, China) and undergoing PCI were consecutively enrolled in the study. The inclusion criteria were as follows: (1) Patients aged 18 years and older; (2) Patients who were diagnosed with ACS confirmed by standard clinical criteria and undergoing PCI; The exclusion criteria were as follows: (1) Patients who diagnosed with stable angina pectoris; (2) Patients who lack critical information, such as the AAR variables and the GRACE score; (3) Patients with a limited life expectancy <1 year; (4) Patients with a history of coronary artery bypass grafting (CABG); (5) Patients who with severe hepatic or renal insufficiency; (6) Patients who died during hospitalization. After strict screening, there were 1498 patients included in the final analyses (**Supplementary Fig. 1**). This retrospective study was thoroughly vetted and granted by the local ethics committee and rigorously complied with the principles of the Declaration of Helsinki, informed consent was obtained from all participants, guaranteeing their awareness and voluntary participation in the research.

### 2.2 Data Definitions and Collection

The electronic medical records were utilized to gather data about sociodemographic characteristics, smoking status, past medical history, GRACE score and each variable of it, AAR, laboratory test results including cardiac enzymes or markers, admission blood glucose (ABG), brain natriuretic peptide (BNP), high-density lipoprotein cholesterol (HDL-C), total cholesterol (TC), triglyceride (TG), and low-density lipoprotein cholesterol (LDL-C) were measured by standard laboratory methods, the left ventricular ejection fraction (LVEF), detailed diagnostic, Angiographic data and Discharge medications (aspirin, P2Y12 receptor inhibitors, statins, β-blockers, angiotensin-converting enzyme inhibitors/angiotensin receptor blockers (ACEIs/ARBs), diuretics, insulin, and oral hypoglycemic agents) of the patients. Past medical history data involved hypertension, previous PCI, atrial fibrillation (AF), DM, chronic obstructive pulmonary disease (COPD), and previous stroke. These medical histories were corroborated by self-reports and medical records. ACS was defined as acute coronary syndrome involving UA, NSTEMI, and STEMI [22, 27]. Hypertension was defined as a diastolic blood pressure (DBP) ≥90 mmHg or a systolic blood pressure (SBP) ≥140 mmHg, in the absence of anti-hypertensive medication [28]. DM was defined as a fasting blood glucose value ≥7 mmol/L, random venous blood glucose value ≥11.1 mmol/L, or hemoglobin A1c (HbA1c) value ≥6.5% and was verified by a 2-hour OGTT venous blood glucose value ≥11.1 mmol/L or the use of antidiabetic medication [29]. The AF, stroke, and COPD were diagnosed by their respective guidelines [30, 31, 32]. During the initial visit, the body weight (kg) and height (m) of each patient were recorded. The body mass index (BMI) was computed as body weight (kg)/height^2^ (m^2^).

Coronary angiography and PCI were conducted by experienced clinicians. All patients underwent targeted vessel revascularization, with the implantation of coronary stents in severely affected coronary arteries exhibiting luminal narrowing exceeding 70%. The severity degree of the coronary artery was evaluated and reflected through the number of stenosed arteries as well as the Synergy between PCI with Taxus and Cardiac Surgery (SYNTAX) score, which were derived from the results of coronary angiography.

Post discharge, following each patient at 1, 6, and 12 months and then every 1 year thereafter. The primary endpoint was MACEs, defined as a composite outcome that encompasses all-cause death, cardiac death, nonfatal myocardial infarction, nonfatal stroke, and unplanned repeated revascularization. The secondary endpoints were analyses of all-cause death, cardiac death, nonfatal myocardial infarction, nonfatal stroke, and unplanned repeated revascularization respectively. All endpoints were rigorously evaluated and documented by experienced physicians and, if necessary, referred to the corresponding medical records for further confirmation.

### 2.3 Computation of the AAR and the GRACE Score

The GRACE risk score was a risk model to predict prognosis in ACS patients [11, 12], computed for every patient upon admission to the hospital based on the following eight clinical parameters: age, history of myocardial infarction, congestive heart failure (CHF), systolic blood pressure, serum creatinine, heart rate, ST-segment depression, cardiac enzymes. The AAR was determined by the formula [admission blood glucose (mg/dL)/albumin (g/L)] or [admission blood glucose (mmol/L) × 18/albumin (g/L)], utilizing blood preparations collected within 24 hours after admission [26], the glucose and albumin were measured by standard laboratory methods.

### 2.4 Statistical Analysis

For quantitative data analysis, data that are normally distributed are typically characterized by means with standard deviations, whereas data that exhibit nonnormal distributions are typically characterized by medians with interquartile ranges. For categorical variables, counts and percentages (%) are usually summarized, and statistical comparisons were computed via the chi-square test or Fisher’s exact test, according to the circumstances. To compare the groups depending on the tertiles of the AAR, the categorical variables were performed via chi-square test, the continuous variables were performed via one-way analysis of variance (ANOVA) (parametric variables) and the Kruskal–Wallis test (nonparametric variables), respectively. The relationship between the AAR and the GRACE risk score was evaluated by Spearman’s rank correlation coefficient. Cox proportional hazard models were employed to identify the independent risk factors correlated with cardiovascular events. Evaluating the predictive value of AAR via Kaplan‒Meier survival curves. The predictive value of the GRACE risk score independently, as well as in conjunction with the AAR, was assessed by the AUC. The added predictive value of incorporating the AAR into the GRACE score was evaluated via C statistics, continuous net reclassification improvement (NRI), and integrated discrimination improvement (IDI). Interior verification utilizing 1000 bootstrap resampling techniques was utilized to evaluate the precision of the prediction models and to mitigate overfitting bias. Furthermore, we recalibrated the model in the verification cohort to substantiate the universality of the improvement in the GRACE risk score through AAR. The utility and net benefit of the model were determined via decision curve analysis. All computations were executed via SPSS version 26.0 software (IBM Corporation, Chicago, IL, USA) and R version 4.0.2 software (R Foundation for Statistical Computing, Vienna, Austria). Statistical significance was confirmed at the *p* < 0.05, utilizing a two-tailed hypothesis test.

## 3. Results

### 3.1 Baseline Characteristics of the Study Population

During a median of 31.25 (27.53, 35.10) months, there were 110 patients lost to follow-up, finally 1498 patients were included in this research. The mean age of the population was 67.19 ± 11.24 years, and there was less female (28.7%). The 1498 individuals were grouped into three groups based on the tertiles of AAR level at admission (T1: AAR <2.45; T2: 2.45 ≤ AAR < 3.30; T3: AAR ≥3.30). The baseline characteristics and demographic data are displayed in Table 1. Among the three groups, there were no significant differences concerning SBP, creatinine level, previous myocardial infarction(MI), female, BMI, smoking status, previous PCI, COPD, hypertension, or previous stroke. However, a comparison across groups revealed that the GRACE score, DM, baseline SYNTAX score (bSS), residual SYNTAX score (rSS), and other risk factors, including age, CHF, AF, cardiac troponin T (cTnT), BNP, ABG, and TG were greater in patients with elevated AAR. Moreover, the high-density lipoprotein (HDL) and LVEF were lower in patients with higher AAR (all *p* < 0.01).

**Table 1.** **Demographic and baseline characteristics according to tertiles of the AAR level**.

Variable		Total (n = 1498)	T1 (n = 498)	T2 (n = 500)	T3 (n = 500)	*p* value
	GRACE variables					
	Age, years	67.19 ± 11.24	65.00 ± 11.29	68.13 ± 11.06	68.43 ± 11.09	<0.001
	SBP, mmHg	132.84 ± 21.06	133.16 ± 18.59	134.30 ± 21.26	131.56 ± 22.26	0.112
	Heart rate, bpm	77.09 ± 14.32	74.75 ± 12.17	76.80 ± 14.08	79.71 ± 16.03	<0.001
	Creatinine, umol/L	77.30 (65.20, 92.33)	76.65 (66.28, 87.53)	77.35 (65.05, 93.05)	78.00 (64.03, 100.50)	0.143
	CHF, n (%)	258 (17.2)	36 (7.2)	80 (16.0)	142 (28.4)	<0.001
	Previous MI, n (%)	70 (4.6)	31 (6.2)	18 (3.6)	21 (4.2)	0.120
	ST-segment depression, n (%)	874 (58.3)	215 (43.2)	323 (64.6)	336 (67.2)	<0.001
	Elevated cardiac enzymes/markers, n (%)	773 (51.6)	177 (35.5)	287 (57.4)	309 (61.8)	<0.001
	GRACE score	104.60 ± 29.66	93.32 ± 25.64	107.01 ± 28.51	113.42 ± 31.01	<0.001
	AAR	3.33 ± 1.66	2.14 ± 0.23	2.81 ± 0.24	5.05 ± 1.86	<0.001
	Female, n (%)	430 (28.7)	129 (25.9)	139 (27.8)	162 (32.4)	0.066
	BMI, kg/m^2^	24.40 ± 3.00	24.27 ± 3.16	24.39 ± 3.09	24.56 ± 3.29	0.416
	Smoking, n (%)	790 (52.7)	275 (55.2)	272 (54.4)	243 (48.6)	0.074
	Previous PCI, n (%)	130 (8.6)	44 (8.8)	43 (8.6)	43 (8.6)	0.988
	COPD, n (%)	82 (5.4)	33 (6.6)	24 (4.8)	25 (5.0)	0.380
	Hypertension, n (%)	1027 (68.5)	331 (66.5)	342 (68.4)	354 (70.8)	0.336
	Diabetes mellitus, n (%)	595 (39.7)	75 (15.1)	147 (29.4)	373 (74.6)	<0.001
	AF, n (%)	115 (7.6)	21 (4.2)	51 (10.2)	43 (8.6)	0.001
	Previous stroke, n (%)	121 (8.0)	30 (6.0)	45 (9.0)	46 (9.2)	0.119
Laboratory measurements						
	cTnT, pg/mL	39.81 (111.84, 666.90)	15.41 (8.54, 117.95)	59.25 (13.51, 1037.00)	122.60 (18.18, 1380.00)	<0.001
	BNP, pg/mL	122.90 (48.60, 341.13)	78.30 (34.70, 190.60)	126.90 (55.40, 345.50)	189.35 (71.78, 603.50)	<0.001
	ABG, mmol/L	7.13 ± 3.33	4.86 ± 0.60	6.03 ± 0.80	10.50 ± 3.82	<0.001
	TG, mmol/L	1.47 (1.07, 2.19)	1.42 (1.04, 1.96)	1.48 (1.08, 2.24)	1.55 (1.01, 2.41)	0.004
	HDL-C, mmol/L	1.12 (0.95, 1.31)	1.17 (1.00, 1.38)	1.11 (0.95, 1.28)	1.09 (0.89, 1.28)	<0.001
	LDL-C, mmol/L	2.61 (2.07, 3.27)	2.57 (2.05, 3.26)	2.65 (2.06, 3.29)	2.65 (2.09, 3.23)	0.566
	TC, mmol/L	4.31 (3.58, 5.18)	4.27 (3.59, 5.21)	4.29 (3.55, 5.18)	4.36 (3.59, 5.17)	0.966
	LVEF, %	54.94 ± 8.91	57.29 ± 7.62	55.05 ± 8.66	52.62 ± 9.67	<0.001
Diagnosis, n (%)						<0.001
	UA	725 (48.3)	321 (64.5)	213 (42.6)	191 (38.2)	
	NSTEMI	433 (28.9)	94 (18.9)	123 (24.6)	123 (24.6)	
	STEMI	340 (22.6)	83 (16.7)	164 (32.8)	186 (37.2)	
Angiographic data						
	Number of stents	1.41 ± 0.87	1.34 ± 0.84	1.44 ± 0.85	1.45 ± 0.92	0.108
	Length of stents, mm	37.05 ± 26.36	35.60 ± 26.03	37.05 ± 25.51	38.49 ± 27.49	0.224
	bSS	13.00 (8.00, 20.00)	10.00 (7.00, 17.25)	14.00 (8.00, 20.00)	15.00 (9.00, 21.50)	<0.001
	rSS	3.00 (0.00, 7.00)	2.00 (0.00, 6.00)	3.00 (0.00, 7.88)	4.00 (0.00, 9.00)	<0.001
Discharge medications						
	Aspirin, n (%)	1458 (97.3)	487 (97.8)	488 (97.6)	483 (96.6)	0.455
	P2Y12 receptor inhibitor, n (%)	1474 (98.3)	487 (97.8)	496 (99.2)	491 (98.2)	0.189
	Statins, n (%)	1465 (97.7)	486 (97.6)	489 (97.8)	490 (98.0)	0.907
	β-blockers, n (%)	1041 (69.4)	328 (65.9)	359 (71.8)	354 (70.8)	0.093
	ACEI/ARB, n (%)	678 (45.2)	245 (49.2)	215 (43.0)	218 (43.6)	0.095
	Diuretics, n (%)	261 (17.4)	49 (9.8)	80 (16.0)	132 (26.4)	<0.001
	Insulin, n (%)	162 (10.8)	13 (2.6)	29 (5.8)	120 (24.0)	<0.001
	Oral hypoglycemic agents, n (%)	408 (27.2)	51 (10.2)	100 (20.0)	257 (51.4)	<0.001

GRACE, the Global Registry of Acute Coronary Events; AAR, admission blood glucose to albumin ratio; HR, heart rate; PCI, percutaneous coronary intervention; SBP, systolic blood pressure; AF, atrial fibrillation; COPD, chronic obstructive pulmonary disease; BNP, brain natriuretic peptide; ABG, admission blood glucose; TG, triglyceride; LDL-C, low-density lipoprotein-C; HDL-C, high-density lipoprotein-C; TC, total cholesterol; UA, unstable angina; NSTEMI, non-ST-segment elevation myocardial infarction; rSS, residual SYNTAX score; STEMI, ST segment elevation myocardial infarction; LVEF, left ventricular ejection fraction; ACEI/ARB, angiotensin-converting enzyme inhibitor/angiotensin receptor blocker; bSS, baseline SYNTAX score; CHF, chronic heart failure; MI, myocardial infarction; BMI, body mass index; cTnT, cardiac troponin T; IQR, interquartile range. Data are presented as the mean ± SD, median (IQR) or n (%).

### 3.2 Comparison of Long-Term Prognosis in Different AAR Groups

Among the total patients, 147 experienced MACEs, 107 experienced all-cause death, 68 experienced cardiac death, 45 experienced myocardial infarction, 131 experienced Repeat revascularization, and 57 experienced stroke (Table 2). Furthermore, comparison across groups revealed that for individuals with a higher AAR, the incidence of MACEs, all-cause death, and cardiac death (*p* < 0.001) was higher. There was a similar trend observed for stroke (*p* = 0.019). In addition, the incidence of myocardial infarction and unplanned revascularization was no significant difference between the cohorts.

**Table 2.** **Differences in the long-term prognosis of patients by AAR**.

Variable	Total (n = 1498)	T1 (n = 498)	T2 (n = 500)	T3 (n = 500)	$\chi^{2}$	*p*
MACEs, n (%)	147 (9.8%)	25 (5.0%)	45 (9.0%)	77 (15.4%)	30.935	<0.001
All-cause death, n (%)	107 (7.1%)	14 (2.8%)	30 (6.0%)	63 (12.6%)	37.522	<0.001
Cardiac death, n (%)	68 (4.5%)	5 (1.0%)	21 (4.2%)	42 (8.4%)	31.694	<0.001
Myocardial infarction, n (%)	45 (3.0%)	12 (2.4%)	17 (3.4%)	16 (3.2%)	0.939	0.625
Repeat revascularization, n (%)	131 (8.7%)	37 (7.4%)	41 (8.2%)	53 (10.5%)	3.422	0.181
Stroke, n (%)	57 (3.8%)	11 (2.2%)	18 (3.6%)	28 (5.6%)	7.925	0.019

The individuals were grouped into three groups based on AAR level at admission (T1: AAR <2.45; T2: 2.45 ≤ AAR < 3.30; T3: AAR ≥3.30). Compared with patients who had lower AAR, the incidence of MACEs, all-cause death, cardiac death and stroke was higher in the higher AAR significantly. MACEs, major adverse cardiac events.

**Fig. 1.**
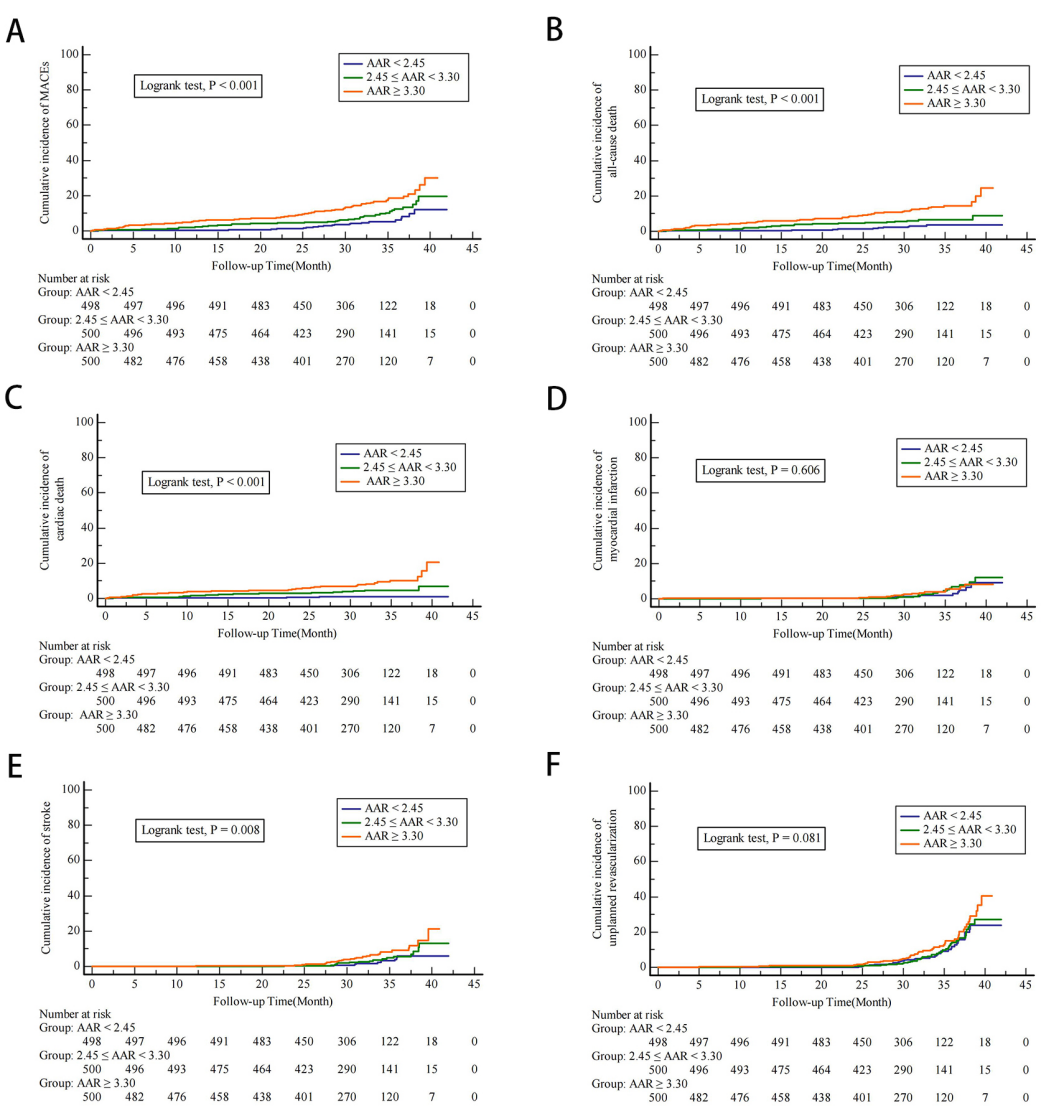
**Cumulative incidence of endpoints according to the groups of AAR**. Kaplan-Meier curves for the cumulative incidence of major adverse cardiac events (MACEs) (A), all-cause death (B), cardiac death (C), nonfatal myocardial infarction (D), nonfatal stroke (E), and unplanned repeat revascularization (F) based on the AAR index tertiles.

As depicted in Fig. 1, the cumulative morbidity of MACEs was higher in patients with higher levels of AAR shown by Kaplan–Meier analysis (log-rank test, *p* < 0.001) (Fig. 1A). This discrepancy may attributed primarily to the increased risks of all-cause death, cardiac death, and nonfatal stroke (Fig. 1B,C,E). However, there were no differences in the myocardial infarction (log-rank test, *p* = 0.606; Fig. 1D) or unplanned revascularization among the different cohorts significantly (log-rank test, *p* = 0.081; Fig. 1F).

Table 3 shows the potential predictive factors for MACEs through univariate and multivariate Cox proportional hazards regression analyses. The univariate analysis indicated that factors such as the GRACE score, female sex, BMI, DM, AAR, rSS, bSS, diuretics, LVEF, and insulin were perceived as latent risk factors for MACEs. Furthermore, evaluating the statistically significant predictive factors was confirmed through univariate screening through multivariate analysis (univariate *p* < 0.05). Following the detection of multicollinearity, multivariate regression analysis showed that both the GRACE score and AAR were independent predictors for MACEs in ACS patients (HR, 1.020; 95% CI: 1.013–1.026; *p* < 0.001; HR, 1.145; 95% CI: 1.045–1.255; *p* = 0.004, respectively).

**Table 3.** **Univariate and multivariate Cox proportional hazards regression analyses for MACEs**.

Variables	Univariate analysis			Multivariate analysis		
	HR	95% CI	*p*	HR	95% CI	*p*
GRACE score	1.026	1.021–1.032	<0.001	1.020	1.013–1.026	<0.001
Female	1.425	1.017–1.995	0.039	1.260	0.892–1.779	0.190
BMI	0.934	0.883–0.988	0.017	0.972	0.920–1.027	0.308
Smoking	0.765	0.553–1.058	0.105			
Previous PCI	1.113	0.652–1.899	0.695			
Hypertension	1.354	0.933–1.965	0.111			
Diabetes mellitus	1.560	1.128–2.155	0.007	1.096	0.732–1.642	0.655
AAR	1.254	1.175–1.339	<0.001	1.145	1.045–1.255	0.004
TC	0.997	0.971–1.023	0.808			
TG	0.906	0.787–1.042	0.165			
HDL-C	0.975	0.796–1.194	0.806			
LDL-C	1.005	0.905–1.117	0.921			
bSS	1.042	1.026–1.059	<0.001	1.009	0.987–1.032	0.433
rSS	1.050	1.029–1.073	<0.001	1.019	0.990–1.048	0.205
LVEF	0.957	0.942–0.972	<0.001	1.001	0.981–1.022	0.924
β-blockers	0.911	0.642–1.293	0.602			
ACEI/ARB	1.073	0.776–1.484	0.669			
Diuretics	3.091	2.206–4.332	<0.001	1.491	0.991–2.244	0.055
Insulin	1.694	1.101–2.605	0.016	0.913	0.548–1.522	0.728

The primary endpoint was MACEs, which is defined as a composite outcome encompassing all-cause death, cardiac death, nonfatal myocardial infarction, nonfatal stroke, and unplanned repeat revascularization.

### 3.3 Incremental Predictive Value of Incorporating the AAR into the GRACE Score

**Fig. 2.**
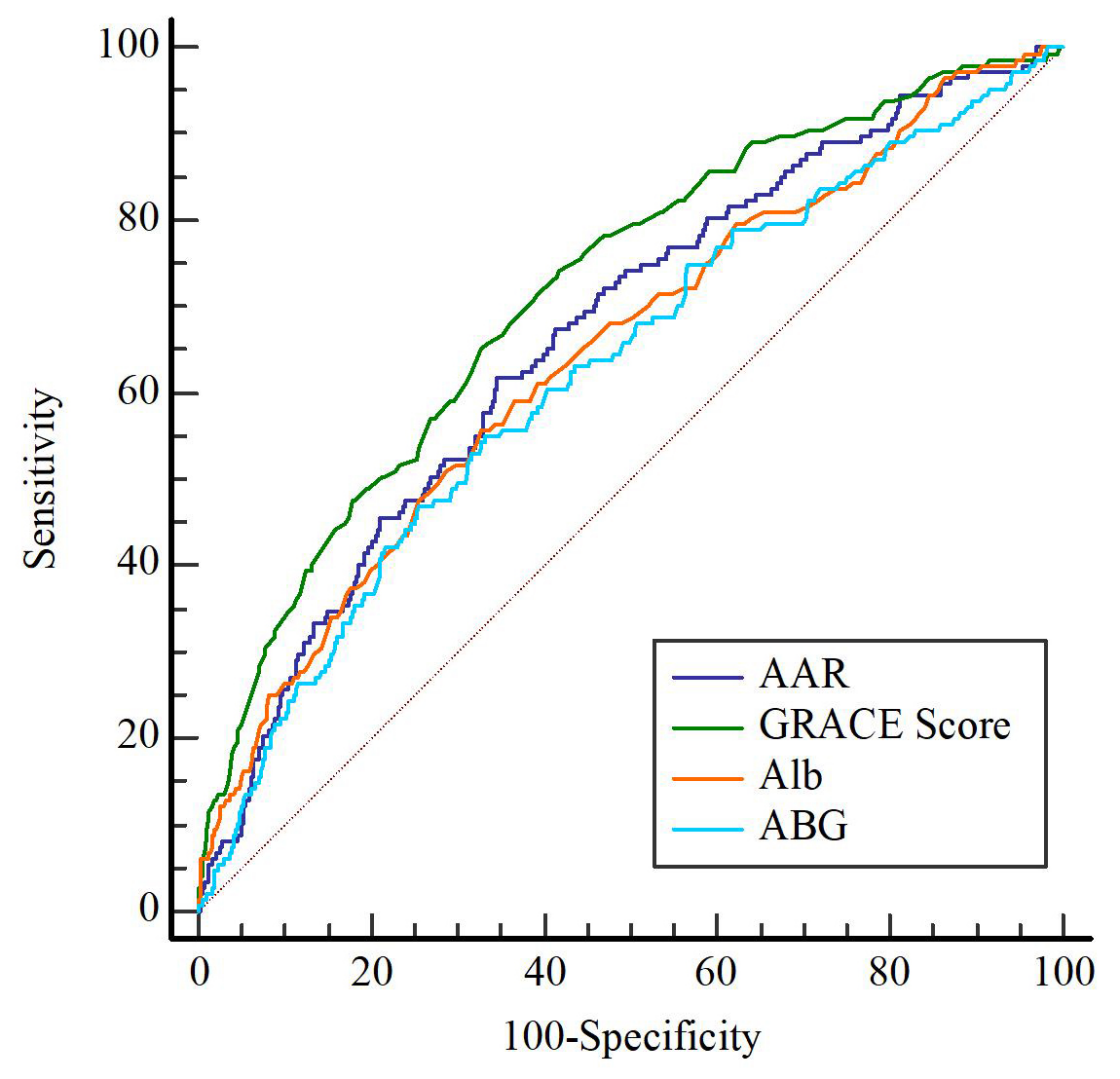
**ROC curve analyses for predicting MACEs**. The AUC of the AAR was 0.665 (95% CI: 0.619–0.711, *p* < 0.001). The AUC of GRACE score was 0.717 (95% CI: 0.673–0.761, *p* < 0.001). The AUC of Alb was 0.645 (95% CI: 0.597–0.694, *p* < 0.001). The AUC of ABG was 0.626 (95% CI: 0.577–0.675, *p* < 0.001). ROC, receiver operating characteristic; AUC, area under the receiver operating characteristic curve.

Since both the GRACE risk score and AAR served as independent risk factors for MACEs, this study further evaluated the predictive efficacy of their combined performance for the long-term prognosis of MACEs. The AUC of the GRACE score, AAR, ABG and Alb for predicting MACEs were 0.717 (95% CI: 0.673–0.761, *p* < 0.001), 0.665 (95% CI: 0.619–0.711, *p* < 0.001), 0.626 (95% CI: 0.577–0.675, *p* < 0.001), and 0.645 (95% CI: 0.597–0.694, *p* < 0.001), respectively (Fig. 2, **Supplementary Table 1**). In the study, we corroborate that the AAR is a superior indicator compared with ABG and albumin for predicting MACEs among patients suffering from ACS after PCI (**Supplementary Table 1**, *p* < 0.001). Further comparisons found that the AUC of the AAR was greater than that of the ABG (**Supplementary Table 2**, *p* < 0.001).

We subsequently assessed the incremental predictive value of incorporating the AAR into the GRACE score (Table 4). Within the deducing cohort, the integration of the AAR substantially increased the C statistic for the prognostication of MACEs, all-cause death, cardiac death, myocardial infarction, stroke, or unplanned repeat revascularization, with increases from 0.717 to 0.733 (*p* < 0.001), 0.726 to 0.757 (*p* < 0.001), 0.775 to 0.813 (*p* < 0.001), and 0.674 to 0.685 (*p* < 0.001) compared with the GRACE score alone, respectively. The consistency of the results was maintained across examinations of the risk models utilizing internal data. Moreover, improvements in discrimination for MACEs, all-cause death, cardiac death, stroke, MI, or repeat revascularization by applying the AAR to the GRACE score were demonstrated by the NRI (0.184, 95% CI: 0.066–0.315, *p* < 0.010; 0.193, 95% CI: 0.043–0.310, *p* < 0.010; 0.272, 95% CI: 0.101–0.409, *p* < 0.010; 0.168, 95% CI: 0.050–0.287, *p* < 0.010) and IDI (0.014, 95% CI: 0.003–0.040, *p* < 0.010; 0.016, 95% CI: 0.003–0.044, *p* < 0.010; 0.017, 95% CI: 0.000–0.047, *p* = 0.030; 0.009, 95% CI: 0.002–0.023, *p* < 0.010). Nonetheless, the incorporation of albumin into the GRACE score did not markedly enhance the IDI or NRI of the novel predictive model (Table 4).

**Table 4.** **The incremental predictive value of incorporating AAR to the GRACE score**.

		C-index (95% CI)	*p*	NRI (95% CI)	*p*	IDI (95% CI)	*p*
MACEs							
	GRACE	0.717 (0.694–0.740)	<0.001	ref	ref	ref	ref
	GRACE+Alb	0.724 (0.701–0.747)	<0.001	0.119 (–0.040–0.248)	0.139	0.005 (–0.001–0.021)	0.159
	GRACE+AAR	0.733 (0.690–0.776)	<0.001	0.184 (0.066–0.315)	<0.010	0.014 (0.003–0.040)	<0.010
Death							
	GRACE	0.726 (0.676–0.776)	<0.001	ref	ref	ref	ref
	GRACE+Alb	0.746 (0.698–0.794)	<0.001	0.158 (0.003–0.261)	0.030	0.007 (–0.002–0.029)	0.129
	GRACE+AAR	0.757 (0.734–0.758)	<0.001	0.193 (0.043–0.310)	0.010	0.016 (0.003–0.044)	<0.010
Cardiac death							
	GRACE	0.775 (0.717–0.833)	<0.001	ref	ref	ref	ref
	GRACE+Alb	0.799 (0.746–0.852)	<0.001	0.155 (–0.021–0.297)	0.090	0.005 (–0.003–0.027)	0.269
	GRACE+AAR	0.813 (0.792–0.832)	<0.001	0.272 (0.101–0.409)	<0.010	0.017 (0.000–0.047)	0.030
Stroke, MI, or revascularization							
	GRACE	0.674 (0.636–0.712)	<0.001	ref	ref	ref	ref
	GRACE+Alb	0.678 (0.641–0.715)	<0.001	0.088 (–0.183–0.193)	0.478	0.001 (–0.001–0.006)	0.388
	GRACE+AAR	0.685 (0.648–0.722)	<0.001	0.168 (0.050–0.287)	<0.010	0.009 (0.002–0.023)	<0.010

The MACEs, which is defined as a composite outcome encompassing all-cause death, cardiac death, MI, stroke, and repeat revascularization. NRI, net reclassification improvement; IDI, integrated discrimination improvement; Alb, albumin. Other abbreviations as shown in Table 1.

### 3.4 Subgroup Analysis

To investigate the consistency of the predictive capability of AAR across diverse demographic cohorts, the enrolled patients were regrouped according to age, sex, smoking, hypertension, diabetes, and acute myocardial infarction (AMI) (**Supplementary Table 3**), however, there were some differences that emerged in different subgroups. The study found that the AAR performed varies in different diabetic states. The AAR was an independent predictor in ACS patients without diabetes (HR 1.567, 95% CI: 1.294–1.898, *p* < 0.001), but not DM. We stratified patients based on 65 years, it shows that although the AAR was not statistically significant in people younger than 65 years, it was correlated with an elevated risk of MACEs among people older than 65 years (HR, 1.203, 95% CI: 1.082–1.338, *p* = 0.001). The same trend was found in nonsmokers (HR 1.164, 95% CI: 1.032–1.3135, *p* = 0.014), hypertension (HR 1.184, 95% CI: 1.070–1.311, *p* = 0.001), and AMI (HR 1.183, 95% CI: 1.061–1.320, *p* = 0.003). It is worth mentioning that higher AAR was correlated with higher risk of MACEs in both male patients (HR 1.154, 95% CI: 1.026–1.297, *p* = 0.017) and female patients (HR, 1.2102; 95% CI: 1.0406–1.4075, *p* = 0.0133), which allowed for an improved universality of the AAR in the population.

**Fig. 3.**
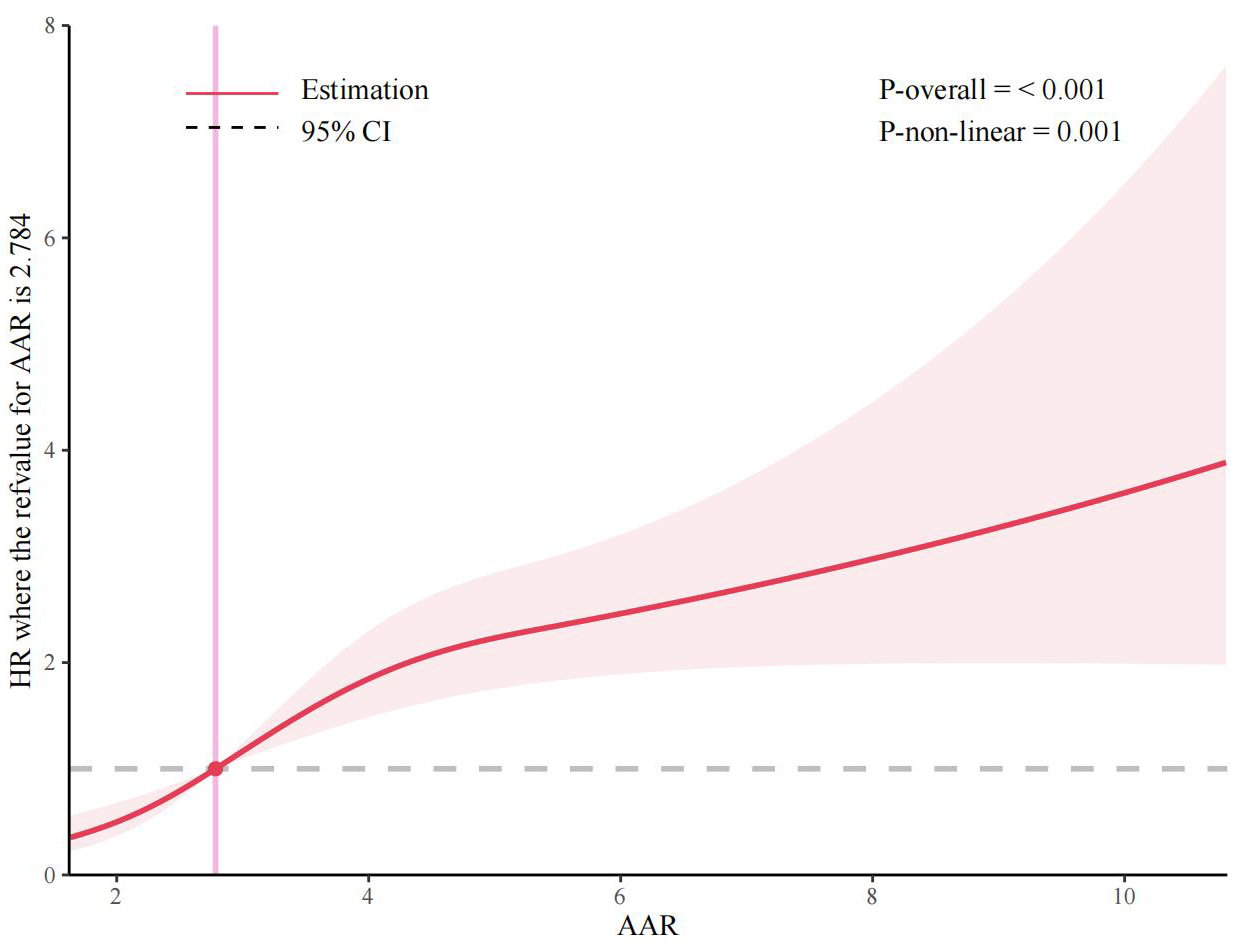
**The Restricted Cubic Splines (RCS) analyses between AAR and MACEs**. The figure shows HR for MACEs adjusted for gender, smoking status, BMI, diabetes mellitus (DM), rSS, LVEF, and the history of medication use for hypertension, DM, and heart failure. Utilizing Cox proportional hazards regression models to fit data. Solid lines indicate HR, and shadow shapes indicate 95% CI.

The Restricted Cubic Spline (RCS) analysis revealed that there was a substantial dose-response relationship between AAR and the incidence of MACEs (Fig. 3). Further investigations revealed a nonlinear correlation between AAR and the occurrence of MACEs (overall model: *p* < 0.001; nonlinear model: *p* = 0.001).

### 3.5 Assessing the Models via Decision Curve Analysis

**Fig. 4.**
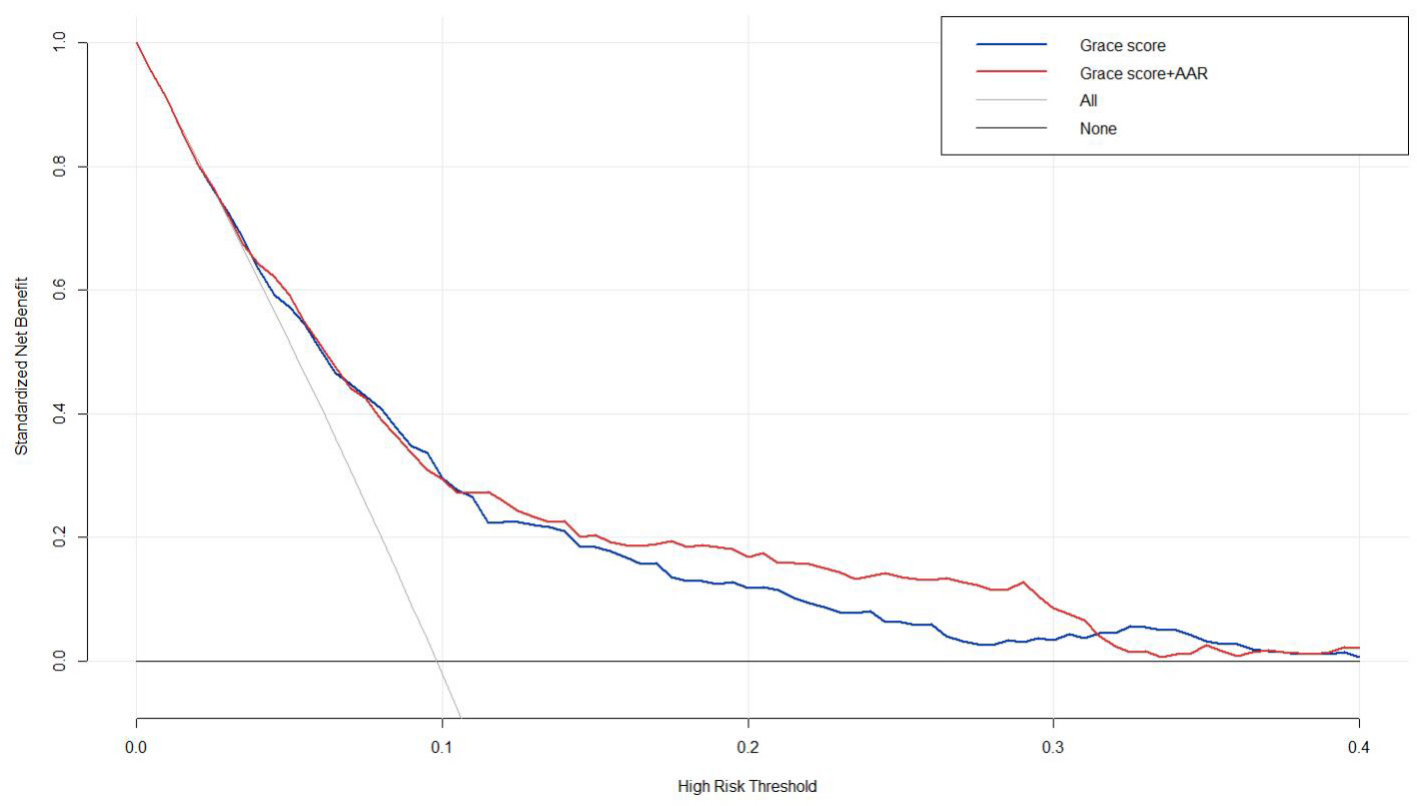
**Decision curve analysis for MACEs**. The clinical utility was compared by decision curve analysis, the x-axis calculates the threshold probability, and the y-axis represents net benefits, which is calculated by subtracting the relative harm (false positives) from the benefits (true positives). Baseline model = Baseline GRACE score; Full model = Adding the AAR to the GRACE score.

Utilizing the decision curve analysis to evaluate the clinical application of the predictive models thoroughly. As depicted in Fig. 4, incorporating the AAR into the GRACE score resulted in a more substantial net benefit across a broad spectrum of long-term prognosis than did the sole application of the basic GRACE score. This finding indicated that integrating the AAR with the GRACE score has clinical utility.

## 4. Discussion

To the best of our knowledge, the present investigation appears to be the first study illustrating that the AAR serves as an independent predictive factor of long-term prognosis among individuals suffering from ACS after PCI. Moreover, the correlation between the AAR and MACEs was more conspicuous among individuals without diabetes. The integration of the AAR into the GRACE score significantly increases its prognostic value in predicting long-term MACEs for discharged patients suffering from ACS.

With advancements in drug treatment and emergency PCI, the mortality rate associated with ACS has significantly decreased compared with that in the past [1], but there are still cardiovascular risks, including those that are currently undetected and inherent, which may lead to adverse prognosis [33]. Early risk stratification is important for improving long-term outcomes for ACS patients. On the one hand, numerous investigations have substantiated the prognostic value of GRACE scores for ACS patients [11, 12]. On the other hand, its predictive value is limited [10, 13, 14], which may be related to the fact that GRACE scores only include electrocardiographic measurements, clinical characteristics, and biochemical parameters upon discharge, and some biomarkers closely related to ACS patients have not been included. Upon admission, both blood glucose and albumin levels serve as easily and rapidly available biomarkers and are strongly correlated with the prognosis of ACS patients. Therefore, the conjunction of AAR to adjust the GRACE score is warranted.

Previous investigations have confirmed that severe fluctuations in blood glucose lead to elevated levels of inflammatory factors, an enhanced oxidative stress response, and impaired endothelial cells, which contribute to the development of atherosclerosis, stress hyperglycemia was an independent predictor of adverse cardiovascular outcomes among ACS patients [34, 35, 36, 37]. Nevertheless, the ABG level is influenced by chronic glycemic status, and it is incapable of revealing the momentary increase in blood glucose values that may occur because of ACS, particularly in patients with DM. Thus, Roberts *et al*. [38] demonstrate that the stress hyperglycemia ratio (SHR) adjusts background blood glucose through HbA1c as a new indicator of relative hyperglycemia serves as an independent predictive factor for ACS patients, which would be a better biomarker of ACS than absolute hyperglycemia [39, 40]. However, HbA1c is not only dependent on glycemic status, but also affected by hemoglobin variants, hereditary hemoglobinopathy, and anemia [41]. Furthermore, HbA1c levels are not always measured especially those without DM [42]. More importantly, for long-term cardiovascular events, the results of SHR were a little inconsistent. There was a study that found SHR played a crucial role in short-period death in patients with AMI but not significantly in long-term mortality [43].

Zhen *et al*. [26] first proposed the AAR index, which was confirmed to be an independent prognosticator for in-hospital all-cause mortality and prognosis after discharge among patients with STEMI after PCI. Similarly, our study verified that the AAR was an independent predictor of adverse prognosis in ACS patients. However, compared with the initial study to analyze AAR by Zhen *et al*. [26], we have some superiority as follows. Firstly, compared to it primarily focused on all-cause mortality in hospitals, our study explored the prognostic value of AAR for long-term outcomes, and respectively analyzed all-cause death, cardiac death, myocardial infarction, stroke, and unplanned revascularization. Secondly, their median follow-up time was just 1.66 years, our study extended it to 31.25 months. Thirdly, the study population was expanded from patients with STEMI to the entire ACS population. However, in our study, the correlation of AAR with adverse outcomes among ACS was not statistically significant in DM patients. The reason may be that AAR relies on absolute hyperglycemia as a molecule and uses the same cut-off values no matter whether DM, but their basic glucose levels were different and the levels of acute elevation under stress varied. Furthermore, DM is characterized by chronic hyperglycemia [43], the DM may itself lead to poor long-term outcomes, resulting in a masked effect of high AAR [44]. In addition, some hyperglycemic patients without a history of DM are truly diabetic patients or prediabetes who are neither diagnosed nor adequately managed, their coronary atherosclerosis and plaque vulnerability are more severe than those of patients without DM, but these conditions are often not detected or ignored [45], would have a greater risk than those with DM [35, 46]. Alternatively, hyperglycaemic patients without DM may have more extensive myocardial damage because of their significantly higher C reactive protein [47]. Moreover, we found that the AAR has a greater predictive value in elderly patients as a poor prognostic marker in ACS patients. There were found that albumin levels are lower and fluctuate more with increasing age, which is closely related to poor prognosis in ACS patients [48, 49]. Furthermore, elderly individuals with a reduction in serum albumin levels, even those within the conventional range, might be at an elevated risk of incident cardiovascular disease [50]. And there has been shown that hyperglycemia is more common in elderly patients, but rarely treated, particularly those without diagnosed diabetes [35].

The etiology of AAR with an adverse prognosis among ACS patients continues to be uncertain and might involve the following mechanisms. First, hyperglycemia may contribute to oxidative stress, platelet activation, endothelial dysfunction, coagulopathy, and restenosis [36, 51, 52], which are correlated with the development and prognosis of cardiovascular disease(CVD). Second, hyperglycemia among ACS patients is correlated with high concentrations of free fatty acids, as well as compromised myocardial glucose metabolism, which in turn augments oxygen consumption and leads to latent deteriorating ischemia [53]. Furthermore, DM is characterized by chronic hyperglycemia, which not only correlates with adverse prognosis in ACS patients but also causes long-term complications like nephropathy, which could decrease serum albumin levels [43]. Albumin is the most extensive protein responsible for binding and transporting a variety of pharmaceutical agents and substances [20]. Human serum albumin has powerful functions, including antioxidant [25] and free radical scavenging [54], inhibition of platelet function [55, 56], and an anticoagulant effect [57]. Low albumin levels often reflect either malnutrition or heightened inflammatory responses [21]. Low serum albumin on admission is not only a trigger for CHD [23], but also closely associated with adverse outcomes in ACS patients [24, 25]. Human serum albumin is susceptible to chemical modifications. In hyperglycemia, the proportion of some chemical modifications increases (such as glycosylation and cysteinylation), leading to decreased levels of albumin [58]. In ACS patients, albumin reacts with reactive oxygen species from free radical damage, hypoxia, or membrane destruction, resulting in ischemia-modified albumin, further reducing serum albumin levels [59]. Moreover, DM hinders albumin synthesis, contributes to albuminuria and reduces the serum albumin level [60, 61]. These mechanisms may provide pathophysiological evidence supporting the outcomes of the current investigation.

The outcomes of the decision curve analysis indicated that combining the GRACE score and the AAR had enhanced predictive ability for high-risk ACS patients compared to the GRACE score in isolation. It is prospected that balance the benefits and potential risks of therapeutic intervention by decision curve analysis, thus guiding physicians to implement more precise treatment strategies of individualized. As an indicator of reactive blood glucose, inflammation, and nutritional status, AAR can more comprehensively reflect the body’s condition than traditional biomarkers such as HbA1c or stress hyperglycemia. It can be easily obtained from the blood routine and liver function, especially suit ACS patients who need rapidly guided emergency treatment. The GRACE score has been widely used in the risk assessment in ACS patients, the addition of AAR provide a better dimension for risk stratification and prediction of long-term prognosis, especially in identifying those who are at traditionally low risk but recently poor glucose control and nutrition. This facilitates superior and refined early risk stratification for patients suffering from ACS, thus enabling patients to receive targeted treatment and management that is more precise and tailored to their needs.

### Strengths and Limitations

The present investigation is potentially the pioneer in evaluating the influence of incorporating the AAR and the GRACE score for predicting the long-term prognosis of ACS patients. Furthermore, the stringent development and verification construction employed in this research serves as a notable advantage, as it prevents potential over-idealization in evaluating the additional predictive value offered by the AAR. Nevertheless, this research has several limitations. The primary constraint of this research is that it is a retrospective observational study, in which data was derived from preexisting records, such as medical records, exposure factors, and self-reported outcomes. These data may be incomplete, missing, or even inaccurate, it is impossible to exclude the influence of other potential factors by randomization. Although we have done our best to reduce these effects through rigorous data screening and statistical analysis, the retrospective nature of the study remains a non-negligible limitation. Secondly, the research was limited to glucose levels at admission, and we were unable to verify the proportion of individuals with elevated glucose upon admission who experienced hyperglycemia throughout their hospitalization. Thirdly, we did not test the subjects during follow-up to determine or exclude a diagnosis of impaired glucose tolerance or diabetes. Finally, the loss-to-follow-up rate is 6.84% (110/1608) in our study. The absence of follow-up data limits our comprehensive assessment of the combined AAR versus GRACE model in predicting more long-term outcomes. To overcome these limitations, we plan to design a multi-center data collection and prospective study design in the future to improve the external validity and accuracy of the study conclusions. Meanwhile, we will actively explore the potential of AAR use in combination with other biomarkers like inflammatory markers and the application of integrated biomarkers in improving GRACE scores to more accurately predict the long-term prognosis of ACS patients and guide more personalized treatment strategies.

## 5. Conclusions

The outcomes of the study indicate that AAR is an independent predictor of long-term unfavorable prognosis in ACS patients. Incorporating AAR into a predictive model with the GRACE score could improve the ability to discern individuals who remain at significant risk for adverse long-term cardiovascular events even after PCI.

## Availability of Data and Materials

The datasets used and/or analyzed during the current study are available from the corresponding author on reasonable request.

## Supplementary Material

Supplementary material associated with this article can be found, in the online version, at https://doi.org/10.31083/RCM26567.
