# Supplementary material for: Incremental Prognostic Value of Admission Blood Glucose to Albumin Ratio in Patients with Acute Coronary Syndrome: A Retrospective Observational Cohort Study
Source: Rev Cardiovasc Med. 2025 Apr 24;26(4):26567. doi: 10.31083/RCM26567 (PMC12059779; doi:10.31083/RCM26567)
Supplement: Supplementary file 1 [file 2153-8174-26-4-26567-s1.docx]

### Supplementary data

**Table of Contents**

**Figures**

**Supplementary Figure 1** 2

**Tables**

**Supplementary Table 1** 3

**Supplementary Table 2** 4

**Supplementary Table 3** 5

**Supplementary Figure 1** Filtrating flow chart of the study population


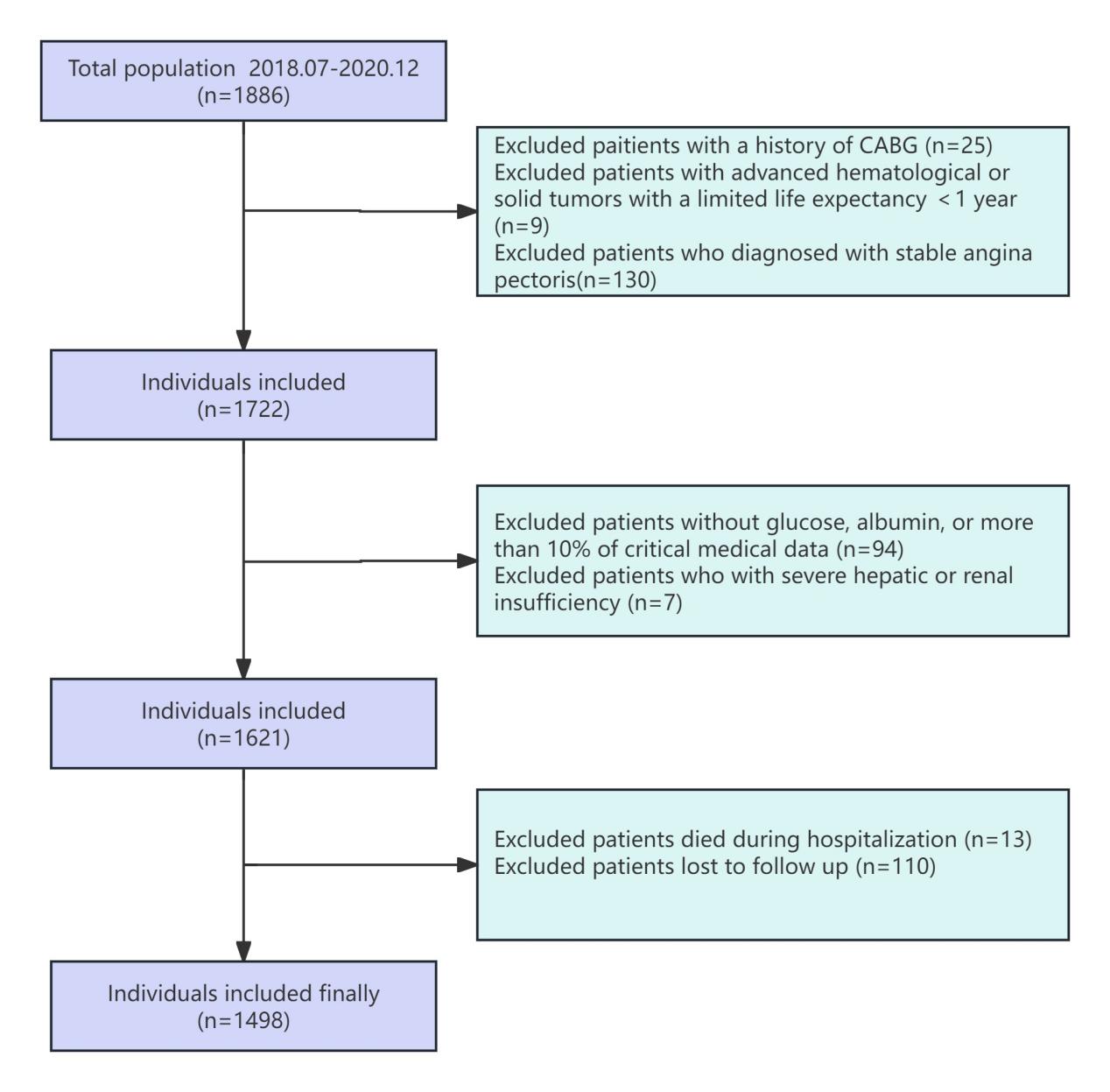


**Supplementary Table 1** ROC curve analysis of the GRACE score, AAR, ABG and Alb for MACEs

| variable | AUC | 95% CI | specificity | sensitivity | P value |
| --- | --- | --- | --- | --- | --- |
| AAR | 0.665 | 0.619-0.711 | 65.43 | 61.90 | < 0.001 |
| GRACE score | 0.717 | 0.673-0.761 | 67.14 | 65.31 | < 0.001 |
| ABG | 0.626 | 0.577-0.675 | 66.69 | 55.10 | < 0.001 |
| Alb | 0.645 | 0.597-0.694 | 67.28 | 55.78 | < 0.001 |

The AUROCs of the GRACE score, AAR, stress hyperglycemia ratio (ABG) and Alb for predicting MACEs were 0.717 (95% CI: 0.673–0.761, P< 0.001), 0.665 (95% CI: 0.619–0.711, P< 0.001), 0.626 (95% CI: 0.577–0.675, P< 0.001), and 0.645 (95% CI: 0.597–0.694, P< 0.001). AAR, admission-blood-glucose-to-albumin ratio; GRACE score, The Global Registry of Acute Coronary Events score; ABG, admission blood glucose; Alb, albumin.

**Supplementary Table 2** The differences between the ROC curves of AAR, ABG, and Alb

|  |  | **Δ**AUC | 95% CI | z statistic | P value |
| --- | --- | --- | --- | --- | --- |
| AAR vs ABG |  | 0.040 | 0.022 to 0.057 | 4.329 | < 0.001 |
| AAR vs Alb |  | 0.020 | -0.036 to 0.076 | 0.702 | 0.483 |

Using ‘roc.compare’ to statistically compare the differences between the AUROC of AAR, ABG, and Alb. The AUROC of the AAR was higher than that of ABG, P< 0.001.

**Supplementary Table 3** Investigating the association between the AAR and the prevalence of MACEs in different subgroups.

| Subgroup | No. of Patients | MACEs | No MACEs | HR(95%CI) | P value |
| --- | --- | --- | --- | --- | --- |
| All patients | 1498 | 147 | 1351 | 1.145 (1.045-1.258) | 0.004 |
| Age |  |  |  |  |  |
| Age≥65years | 918 | 115(12.5%) | 803(87.5%) | 1.203 (1.082-1.338) | 0.001 |
| Age＜65years | 580 | 32(5.5%) | 115(94.5%) | 0.927 (0.748-1.149) | 0.487 |
| Sex |  |  |  |  |  |
| Male | 1068 | 94(8.8%) | 974(91.2%) | 1.154 (1.027-1.298) | 0.017 |
| Female | 430 | 53(12.3%) | 377(87.7%) | 1.210 (1.041-1.408) | 0.013 |
| Smoking |  |  |  |  |  |
| Yes | 790 | 68(8.6%) | 722(91.4%) | 1.149 (0.995-1.328) | 0.060 |
| No | 708 | 79(11.2%) | 629(88.8%) | 1.164 (1.032-1.313) | 0.014 |
| Hypertension |  |  |  |  |  |
| Yes | 1027 | 110(10.7%) | 917(89.3%) | 1.184 (1.070-1.311) | 0.001 |
| No | 471 | 37(7.9%) | 434(92.1%) | 0.948 (0.760-1.182) | 0.636 |
| Diabetes mellitus |  |  |  |  |  |
| Yes | 595 | 74(12.4%) | 521(87.6%) | 1.067 (0.958-1.187) | 0.238 |
| No | 903 | 73(8.1%) | 830(91.9%) | 1.567 (1.294-1.898) | <0.001 |
| AMI |  |  |  |  |  |
| Yes | 773 | 93(12.0%) | 680(88.0%) | 1.183 (1.061-1.320) | 0.003 |
| No | 725 | 54(8.4%) | 671(92.6%) | 1.134 (0.943-1.365) | 0.182 |

The participants were regrouped according to age, sex, smoking, hypertension, diabetes, and AMI, to investigate the consistency of the predictive capability of AAR across diverse demographic cohorts. The abbreviations as shown in Table 1.
